# Supplementary material for: Content-rich biological network constructed by mining PubMed abstracts
Source: BMC Bioinformatics. 2004 Oct 8;5:147. doi: 10.1186/1471-2105-5-147 (PMC528731; doi:10.1186/1471-2105-5-147)
Supplement: Additional File 2 — The original results of the above study (non-essential files are deleted to keep the file size under the limit set by BMC bioinformatics). [file 1471-2105-5-147-S2.bz2 › chilibotAdditionalFile2/dip05/645ID11060015E11032/html/CRR_PTSH.html]

 


 **CRR** and **PTSH** 
  
Found 24 abstracts in PubMed, retrieved 05.  
 

 What does Google say? 
 PDF only 
| .edu only 

---

**Interactive relationship** (e.g. stimulation, inhibition, etc)

**Non-interactive relationship** (e.g. studied together, co-existance, homology, etc.)

- Expression plasmids were constructed which contained the genes encoding enzyme I ptsI, HP  **ptsH** , IIAGlc  **crr** , and IICBGlc ptsG of the glucose phosphotransferase system behind inducible promoters.  Ref: 7601098 Eur J Biochem, 1995
- In contrast, we successfully identified three phosphotransferase system genes,  **ptsH** , ptsI, and  **crr**  in Buchnera, and provide evidence for their active expression.  Ref: 10467175 J Biochem Tokyo , 1999
- Three genes encoding homologues of the general PTS components enzyme I ptsI, HPr  **ptsH** , and enzyme IIA  **Crr**   **crr** .  Ref: 11386346 Antonie Van Leeuwenhoek, 2000
- BACKGROUND The pts operon of Escherichia coli consists of three genes  **ptsH** , ptsI and  **crr** , each encoding for central components of the phosphoenolpyruvate carbohydrate phosphotransferase system, HPr, enzyme I and IIAGlc, respectively.  Ref: 10469172 Genes Cells, 1999
